# miR-133a-3p and miR-145-5p co-promote goat hair follicle stem cell differentiation by regulating NANOG and SOX9 expression

**DOI:** 10.5713/ab.23.0348

**Published:** 2023-11-02

**Authors:** Jian Wang, Xi Wu, Liuming Zhang, Qiang Wang, Xiaomei Sun, Dejun Ji, Yongjun Li

**Affiliations:** 1 Key Laboratory of Animal Genetics & Molecular Breeding of Jiangsu Province, College of Animal Science and Technology, Yangzhou University, Yangzhou 225009, China; 2 International Joint Research Laboratory in Universities of Jiangsu Province of China for Domestic Animal Germplasm Resources and Genetic Improvement, Yangzhou University, Yangzhou 225009, China

**Keywords:** miR-133a-3p, miR-145-5p, NANOG, SOX9, Stem Cell Differentiation

## Abstract

**Objective:**

Hair follicle stem cells (HFSCs) differentiation is a critical physiological progress in skin hair follicle (HF) formation. Goat HFSCs differentiation is one of the essential processes of superior-quality brush hair (SQBH) synthesis. However, knowledge regarding the functions and roles of miR-133a-3p and miR-145-5p in differentiated goat HFSCs is limited.

**Methods:**

To examine the significance of chi-miR-133a-3p and chi-miR-145-5p in differentiated HFSCs, overexpression and knockdown experiments of miR-133a-3p and miR-145-5p (Mimics and Inhibitors) separately or combined were performed. NANOG, SOX9, and stem cell differentiated markers (β-catenin, C-myc, Keratin 6 [KRT6]) expression levels were detected and analyzed by using real-time quantitative polymerase chain reaction, western blotting, and immunofluorescence assays in differentiated goat HFSCs.

**Results:**

miR-133a-3p and miR-145-5p inhibit NANOG (a gene recognized in keeping and maintaining the totipotency of embryonic stem cells) expression and promote SOX9 (an important stem cell transcription factor) expression in differentiated stem cells. Functional studies showed that miR-133a-3p and miR-145-5p individually or together overexpression can facilitate goat HFSCs differentiation, whereas suppressing miR-133a-3p and miR-145-5p or both inhibiting can inhibit goat HFSCs differentiation.

**Conclusion:**

These findings could more completely explain the modulatory function of miR-133a-3p and miR-145-5p in goat HFSCs growth, which also provide more understandings for further investigating goat hair follicle development.

## INTRODUCTION

Animals’ hair growth is tightly associated with skin hair follicle (HF) formation, and the differentiation of cells in skin hair follicles is critical for hair follicle development, especially the differentiation of hair follicle stem cells [1–3]. Hair follicle stem cells (HFSCs) are located within the outer root sheaths of hair follicles (HF), which are capable of self-renewal, maintaining undifferentiated ability, and have proliferative capacity *in vivo* and *in vitro*. HFSCs are particularly physiological value in medicine and biology owing to their self-renewal and differentiation activities [4]. NANOG, a unique homeobox transcription factor, is one of the key downstream effectors in Wnt and BMP signals [5]. NANOG, is also a novel master gene of embryonic stem cell (ESC) pluripotency, which plays vital roles in controlling the pluripotent inner cell mass during embryonic development, sustaining the pluripotent epiblast, and while suppressing primitive endoderm differentiation [6]. Besides, NANOG is ubiquitously found in the vigorous divided cells, and as the cell differentiation degree deepens, the expression level of NANOG gradually decreases until it cannot be detected in fully differentiated cells [7,8]. SOX9, an important transcription factor, regulates multiple cell type formation, the development of hair follicle (HF), inner ear, testis, heart, kidney, and central nervous system. SOX9 is also a direct modulator of Activin/transforming growth factor beta (TGFβ) signaling related genes [9,10]. However, the regulation effect of NANOG and SOX9 in goat HFSCs remains unclear.

Hair follicle development and hair formation are associated with important economic value for commercial applications, such as, Cashmere. Yangtze River Delta white goats’ (YRDWG) HFSCs are harvested from newborn male’s goat neck skin [11]. Our previous research revealed that i) goat HFSCs differentiation is indispensable for superior-quality brush hair (SQBH) formation, this hair is a popular product for its vivid white color, magnificent luster and fine elastic properties, which only can be harvested from YRDWG and is the finest raw material for making Chinese calligraphy brushes [12,13]. ii) β-catenin, C-myc, KRT6 can function as the positive regulators of goat HFSCs differentiation, and they were high expression in differentiated stem cells [14]. β-Catenin participates in the Wnt/β-catenin axis to activate the downstream effector C-myc and Bmp4 levels, and further inducing HFSCs differentiation. KRT6, a member protein in keratins family, is an important HFSCs differentiation marker [15,16]. These studies revealed that β-catenin, C-myc, KRT6 are essential markers and regulatory factors which can influence goat HFSCs differentiation.

Noncoding RNA, including miRNAs, lncRNAs, circRNAs etc., have been gradually revealed and recognized to be associated with the control of skin HF development. Namely, miR-214 [17], miR-218-5p [18], lncRNA-lncRNA5322 [19], lncRNA-PlncRNA-1 [20], circRNA-0100 [21], and circRNA-1926 [22], which are essential for skin HF development (HFSCs proliferation and differentiation) and regeneration, cashmere goat secondary HFSCs into HF lineage. Furthermore, in prior research, we demonstrated that miR-149-5p positively regulate goat HFSCs proliferation and differentiation [14,23], circCOL1A1 sequesters miR-149-5p and controls the CMTM3/AR axis, to further suppresses goat HFSCs proliferation, differentiation, and HF development [24]. miR-133a-3p and miR-145-5p together target DUSP6 to further goat HFSCs proliferation and growth [25,26]. However, knowledge regarding the separate and conjoint functions of miR-133a-3p and miR-145-5p during skin HFSCs differentiation in YRDWG is limited.

Here, we discovered the modulatory function of miR-133a-3p and miR-145-5p in goat HFSCs differentiation. We determined that miR-133a-3p and miR-145-5p could suppress the goat HFSCs multipotency by inhibiting the pluripotent gene NANOG and promoting the stem cell differentiation regulatory factor SOX9 expression; miR-133a-3p and miR-145-5p could promote goat HFSCs differentiation via upregulation of β-catenin, C-myc, KRT6; miR-133a-3p and miR-145-5p knockdown in the differentiated stem cells repressed β-catenin, C-myc, KRT6 expression. These findings of this study could explain the modulatory function of miR-133a-3p and miR-145-5p in goat HFSCs differentiation and goat HF development.

## MATERIALS AND METHODS

### Animal care

The animals, materials and experimental procedures used in this study were approved by the Institutional Animal Care and Use Committee, Yangzhou University, Yangzhou, China (Approval ID: SYXK [Su] 2021-0026, number: 202301020).

### Cell culture

HFSCs were extracted and cultured from the newborn Yangtze River Delta white goats (YEDWG) neck skin as reported in our prior investigation [11,23], and briefly described below: i) Skin tissues were washed with 0.9% normal saline followed by 75% ethanol with 1% penicillin-streptomycin (Invitrogen, Carlsbad, CA, USA) three times. ii) Tissues were then rinsed with phosphate buffered saline (PBS) (Solarbio, Beijing, China) three times and cut into small pieces (approximately 1 mm^3^). iii) Digestion was performed with 0.25% Trypsin-ethylenediaminetetraacetic acid disodium salt (EDTA) (Gibco, New York, USA) at 37°C for 1.5 h. iv) After digestion, HFs were picked and harvested by means of a stereomicroscope (Leica, Wetzlar, Germany). v) The harvested hair follicles were digested with 0.25% Trypsin-EDTA again at 37°C for 30 min. vi) The digested follicles were placed in Dulbecco’s modified eagle medium (DMEM)-F12 supplemented with 20% fetal bovine serum (FBS) and 2% penicillin-streptomycin and were ground in a homogenizer. vii) Finally, the mixed medium was filtered through a 200-mesh cell strainer (Corning, New York, USA) and cultured in 60 mm culture plates at 37°C. Based on this description, we grew HFSCs in 6-well plates (Corning, USA) in 2 mL growth medium consisting DMEM-F12 (Gibco, USA), 20% FBS (Gibco, USA) and 2% penicillin-streptomycin (Invitrogen, USA) with incubation at 37°C in 5% CO_2_. All research protocols received ethical approval from the Yangzhou University.

### Synthesis of RNA oligos

The chi-miR-133a-3p and chi-miR-145-5p oligos, namely, mi-NC (mimics [Mc] negative control, which serving as miR-133a-3p and miR-145-5p Mc inner control), miR-133a-3p Mc, miR-145-5p Mc, anti-NC (the inner control of miR-133a-3p and miR-145-5p inhibitors), miR-133a-3p inhibitors and miR-145-5p inhibitors were designed and acquired from Gene Pharma (Suzhou, China), and summarized in Table 1.

### Cell incorporation

According to our previous study, a β-catenin gene overexpression vector was utilized to stimulate HFSCs differentiation [14]. Oligos incorporation was initiated once the stably overexpression β-catenin stem cells reached ~70% to 80% confluency. The miR-133a-3p and miR-145-5p oligos were independently incorporated into the stem cells. Subsequently, the HFSCs were grown, and differentiation was stimulated for up to 5 days. All HFSCs cultures were done in triplicate or higher.

### RNA extraction, reverse transcription polymerase chain reaction (RT-PCR), and real-time quantitative polymerase chain reaction (RT-q-PCR)

Total RNA extraction was completed from differentiated HFSCs with using a TRIzol (Takara, Japan). To quantity chi-miR-133a-3p and chi-miR-145-5p, we performed reverse transcription with a stem-loop RT-primer and oligo-primers (dT-F17). To assess gene expression, we employed a Takara Prime-Script RT kit accompanied a gDNA eraser (Tokyo, Japan) to reverse-transcribe total RNA into cDNA. All real-time quantitative polymerase chain reaction (RT-q-PCR) reactions were conducted thrice and employed the ABI 7500/7500-Fast real-time PCR system (Applied Biosystems, CA, USA) with a Takara TB Green II Master Mix Reagent Kit (Tokyo, Japan). The 18S RNA (for the chi-miR-133a-3p and chi-miR-145-5p quantification) and glyceraldehyde-3-phosphate dehydrogenase (GAPDH) served as the normalization control. The chi-miR-133a-3p, chi-miR-145-5p and genes relative abundances were computed with the 2^−ΔΔCt^ formula [27,28]. The miRNA and protein-coding gene primers are summarized in Table 2.

### Western blotting analysis

Total protein extraction employed 1% phenylmethanesulfonylfluoride (Solarbio, China)-supplemented radio immunoprecipitation assay lysis buffer (Solarbio, China), and quantification utilized the bicinchoninic acid protein assay kit (Solarbio, China). Subsequently, 20 μg protein was electrophoresed onto 7.5% or 12.5% sodium dodecyl sulfate-polyacrylamide electrophoresis gels, prior to transfer to polyvinylidene fluoride (PVDF) membranes (Immobilon, Darmstadt, Germany).

Thereafter, membranes underwent blocking in 5% skim milk (Sangon Biotech, Shanghai, China), prior to an overnight (ON) incubation at 4°C in primary antibodies against NANOG, SOX9, β-catenin, C-myc, KRT6 and β-actin, including NANOG (CN: ab109250, 1:2,000 dilution), SOX9 (CN: ab185966, 1:2,000 dilution), β-catenin (CN: ab32572, 1:5,000 dilution), C-myc (CN: ab32072, 1:1,000 dilution), KRT6 (CN: ab93279, 1:2,000 dilution) and β-actin (CN: ab8229, 1:500 dilution). All antibodies were purchased from Abcam, Cambridge, United Kingdom.

This was followed by gentle rinsing and additional incubation in horseradish peroxidase-conjugated secondary antibodies (namely, goat-specific anti-rabbit IgG, and rabbit-specific anti-goat IgG; Bio-world, Nanjing, China; CN: BS13278, CN:BS30503, 1:5,000 dilution). Protein visualization employed a Super-Enhanced ECL Reagent (Biosharp, Hefei, China), analysis and quantification utilized Bio-Rad ChemiDoc Touch Imaging System and Image Lab (v5.2.1) (both Bio-Rad, Hercules, CA, USA), respectively.

### Immunofluorescence assay

HFSCs were differentiated for 5 days using distinct treatments, and prior to three PBS rinse, 30-min 4% paraformaldehyde-based fixation at room temperature (RT) and a 15-min permeabilization using PBS with 0.5% Triton X-100 reagent and then blocked using quick block blocking buffer (Beyotime, Shanghai, China). This was followed by an ON incubation at 4°C with anti-β-catenin (1:100 dilution), anti-C-myc (1:100 dilution), and anti-KRT6 (1:50 dilution) immunofluorescence (IF) staining antibodies, followed by a 1-h incubation in goat anti-rabbit IgG H&L (Alexa Fluor 488) secondary antibodies (1:200 dilutions) at RT. All IF antibodies were also purchased from Abcam, Cambridge, United Kingdom.

Following Hoechst-33342 (Beyotime, China) nuclear staining, and three times of PBS rinse in the dark, prior to fluorescent images capture via a Leica fluorescence microscope (Leica, Germany). IF quantification employed the ImageJ software. Lastly, integrated optical density (IOD) was computed by dividing the total IOD by the area.

### Statistical analysis

Data are provided as a mean of 3 replicates±standard error of the mean. Inter-group (NC vs miR-133a-3p, miR-145-5p Mc and double-Mc; anti-NC vs miR-133a-3p, miR-145-5p inhibitors and double-inhibitors) comparisons were assessed using two-independent-samples t-tests in SPSS v24 and Origin(R) 2022 software (OriginLab, Northampton, MA, USA). p-values<0.05 were adjusted as the significant threshold, and p-values<0.01 were regarded as exceedingly significant. * p<0.05 and ** p<0.01.

## RESULTS

### chi-miR-133a-3p and chi-miR-145-5p suppress goat HFSCs pluripotency

To examine the significance of chi-miR-133a-3p and chi-miR-145-5p in differentiated HFSCs, overexpression and knockdown experiments of miR-133a-3p and miR-145-5p separately or combined were performed. The efficacy of miR-133a-3p and miR-145-5p oligos was primarily performed in the stem cells. miR-133a-3p and miR-145-5p expression was significantly increased or inhibited by transfection with Mc and inhibitors for 5 days (Supplementary Figure S1A and 1B).

**Figure 1:**
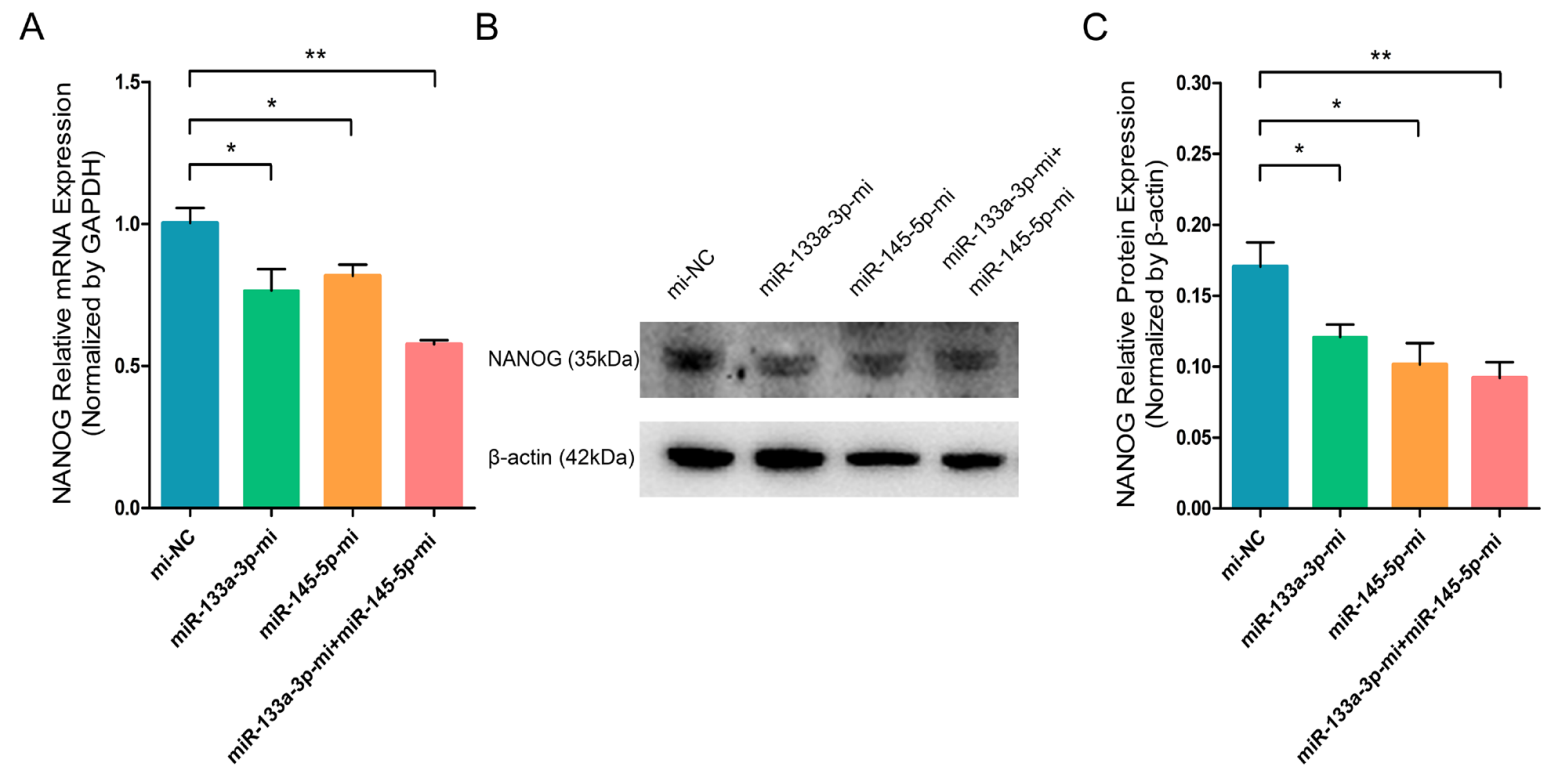
miR-133a-3p and miR-145-5p mimics (Mc) inhibit the NANOG expression in differentiated hair follicle stem cells (HFSCs). (A) Relative NANOG mRNA NANOG 5 days post miR-133a-3p and miR-145-5p mimics (Mc) incorporation. (B, C) Relative NANOG protein expression 5 days post miR-133a-3p and miR-145-5p mimics (Mc) incorporation. * p<0.05; ** p<0.01.

**Figure 2:**
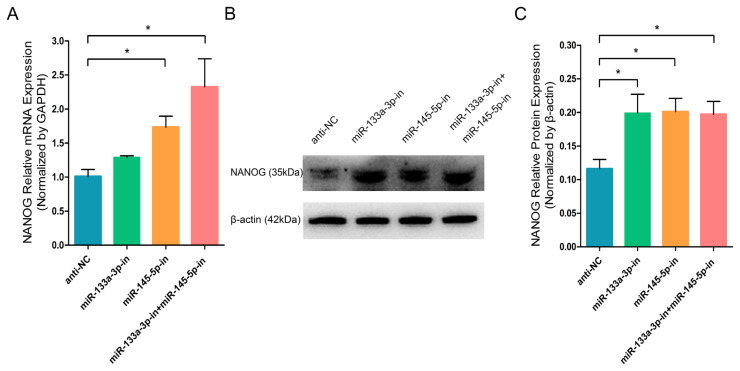
miR-133a-3p and miR-145-5p inhibitors enhance NANOG expression in differentiated hair follicle stem cells (HFSCs). (A) Relative NANOG mRNA expression 5 days following miR-133a-3p and miR-145-5p inhibitors incorporation. (B, C) Relative NANOG protein expression 5 days following miR-133a-3p and miR-145-5p inhibitors incorporation. * p<0.05.

Then, we collected RNA and protein from differentiated stem cells for RT-q-PCR and WB analyses to evaluate the content of the crucial stem cell self-renewing factor and the marker gene of stem cell pluripotency, NANOG. RT-q-PCR and WB analyses revealed that miR-133a-3p and miR-145-5p separately or combined overexpression significantly reduced the mRNA (Figure 1A) and protein (Figure 1B, 1C) expression of NANOG. By contrast, miR-133a-3p and miR-145-5p separately or combined inhibition markedly enhanced the mRNA (Figure 2A) and protein (Figure 2B, 2C) expression of NANOG. These results indicated that miR-133a-3p and miR-145-5p reduce NANOG expression, which might suppress the pluripotency of goat hair follicle stem cells.

### chi-miR-133a-3p and chi-miR-145-5p facilitate the transcription factor content in goat HFSCs

**Figure 3:**
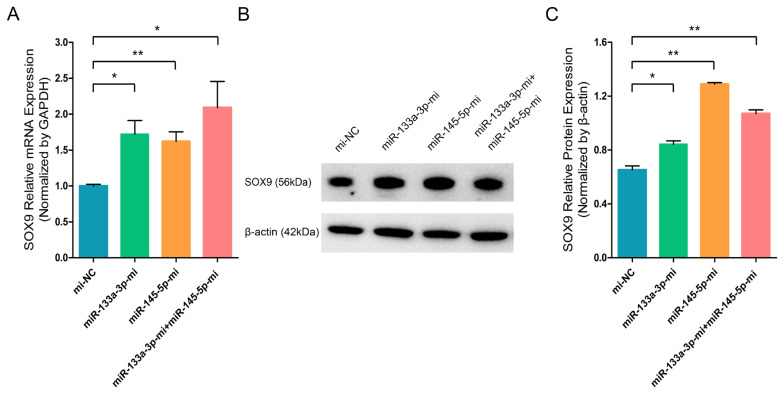
miR-133a-3p and miR-145-5p mimics (Mc) promote SOX9 expression in differentiated hair follicle stem cells (HFSCs). (A) Relative SOX9 mRNA levels 5 days post miR-133a-3p and miR-145-5p mimics (Mc) incorporation. (B, C) Relative SOX9 protein expression 5 days post miR-133a-3p and miR-145-5p mimics (Mc) incorporation. * p<0.05; ** p<0.01.

**Figure 4:**
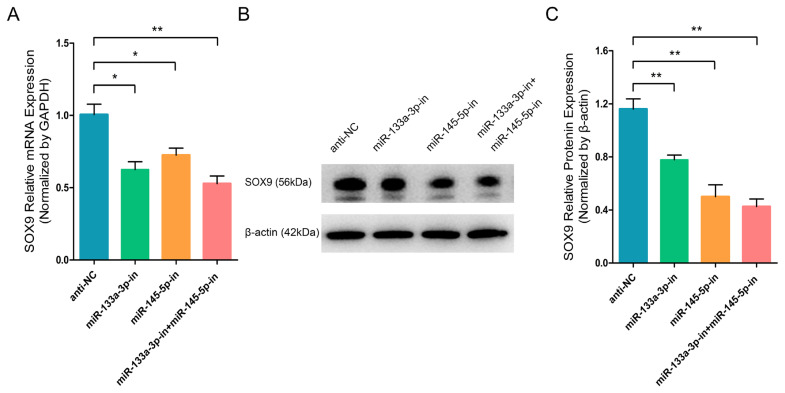
miR-133a-3p and miR-145-5p inhibitors inhibit the SOX9 expression in differentiated hair follicle stem cells (HFSCs). (A) Relative SOX9 mRNA expression 5 days following miR-133a-3p and miR-145-5p inhibitors incorporation. (B, C) Relative SXO9 protein expression 5 days following miR-133a-3p and miR-145-5p inhibitors incorporation. * p<0.05; ** p<0.01.

To further investigate the effect of chi-miR-133a-3p and chi-miR-145-5p on the critical transcription factor and stem cell differentiation regulatory factor SOX9 in goat HFSCs. RT-q-PCR and WB assays showed that miR-133a-3p and miR-145-5p separately or combined overexpression clearly promoted the mRNA (Figure 3A) and protein (Figure 3B, 3C) expression of *SOX9* gene. While, miR-133a-3p and miR-145-5p separately or combined inhibition strongly inhibited SOX9 transcript (Figure 4A) and protein (Figure 4B, 4C). In contrast to *NANOG* gene, miR-133a-3p and miR-145-5p facilitated stem cell differentiation regulatory factor SOX9 expression in goat HFSCs.

### chi-miR-133a-3p and chi-miR-145-5p promote goat HFSCs differentiation

**Figure 5:**
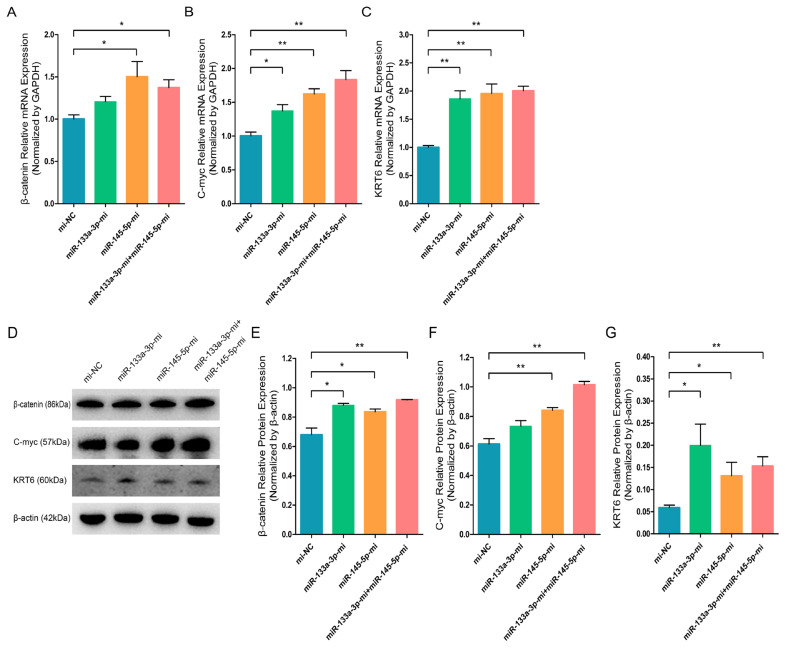
miR-133a-3p and miR-145-5p facilitate the expression of differentiation bio-markers in hair follicle stem cells (HFSCs). (A) Relative β-catenin mRNA content 5 days following miR-133a-3p and miR-145-5p mimics (Mc) incorporation. (B) Relative C-myc mRNA content 5 days following miR-133a-3p and miR-145-5p mimics (Mc) incorporation. (C) Relative KRT6 mRNA content 5 days following miR-133a-3p and miR-145-5p mimics (Mc) incorporation. (D, E) Relative β-catenin protein content 5 days following miR-133a-3p and miR-145-5p mimics (Mc) incorporation. (D, F) Relative C-myc protein 5 days following miR-133a-3p and miR-145-5p mimics (Mc) incorporation. (D, G) Relative KRT6 protein content 5 days following miR-133a-3p and miR-145-5p mimics (Mc) incorporation. * p<0.05; ** p<0.01.

Since our previous findings revealed that chi-miR-133a-3p and chi-miR-145-5p suppress the NANOG expression and facilitate SOX9 expression during goat HFSCs differentiation. Therefore, we speculated that miR-133a-3p and miR-145-5p may play important roles during goat HFSCs differentiation. Then, to explore the function significance of miR-133a-3p and miR-145-5p in stem cell differentiation, we separately transfected Mc-NC, miR-133a-3p Mc, miR-145-5p Mc and co-transfected miR-133a-3p Mc and miR-145-5p Mc into HFSCs and further incubated them for 5 days. Based on our RT-q-PCR data, miR-133a-3p Mc, miR-145-5p Mc, miR-133a-3p Mc+miR-145-5p Mc enhanced HFSCs differentiation regulators, such as, β-catenin (Figure 5A), C-myc (Figure 5B), KRT6 (Figure 5C) transcript levels. Using WB, we demonstrated that β-catenin (Figure 5D, 5E), C-myc (Figure 5D, 5F), KRT6 (Figure 5D, 5G) protein expressions were strongly enhanced in miR-133a-3p Mc, miR-145-5p Mc and miR-133a-3p Mc + miR-145-5p Mc treated groups. These findings primarily suggested that miR-133a-3p and miR-145-5p overexpression potentially stimulates goat HFSCs differentiation.

### Inhibition of chi-miR-133a-3p and chi-miR-145-5p represses goat HFSCs differentiation

**Figure 6:**
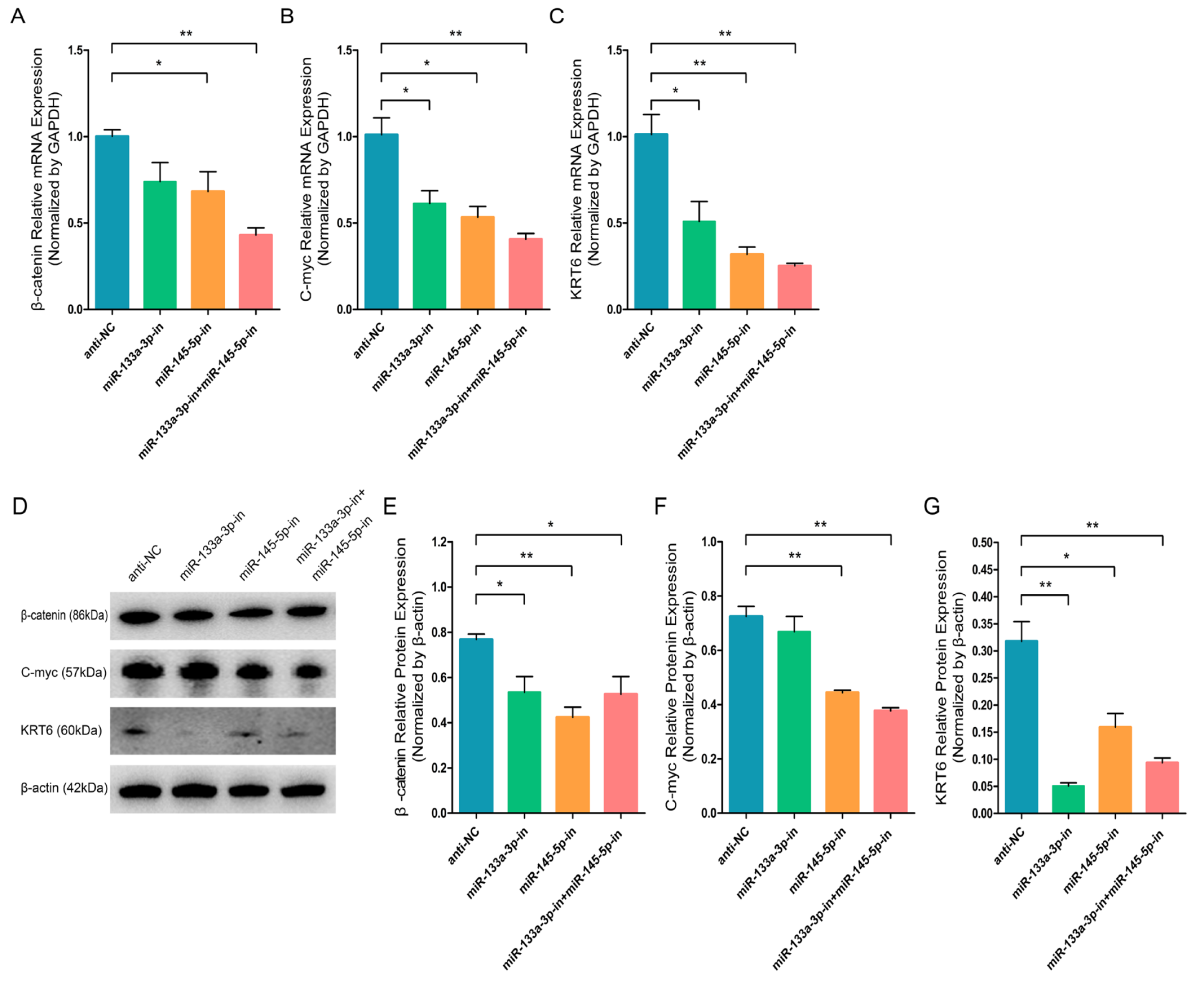
miR-133a-3p and miR-145-5p inhibitors suppress differentiation bio-markers in hair follicle stem cells (HFSCs). (A) Relative β-catenin mRNA levels 5 days following miR-133a-3p and miR-145-5p inhibitors incorporation. (B) Relative C-myc mRNA levels 5 days following miR-133a-3p and miR-145-5p inhibitors incorporation. (C) Relative KRT6 mRNA levels 5 days following miR-133a-3p and miR-145-5p inhibitors incorporation. (D, E) Relative β-catenin protein levels 5 days following miR-133a-3p and miR-145-5p inhibitors incorporation. (D, F) Relative C-myc protein levels 5 days following miR-133a-3p and miR-145-5p inhibitors incorporation. (D, G) Relative KRT6 protein levels 5 days following miR-133a-3p and miR-145-5p inhibitors incorporation. * p<0.05; ** p<0.01.

To ulteriorly elucidate the significant roles of miR-133a-3p and miR-145-5p in goat HFSCs differentiation, we separately transfected anti-NC, miR-133a-3p inhibitors, miR-145-5p inhibitors and co-transfected miR-133a-3p inhibitors and miR-145-5p inhibitors into HFSCs and further incubated them for 5 days. In contrast to overexpression of miR-133a-3p and miR-145-5p, miR-133a-3p, and miR-145-5p knockdown strongly inhibited β-catenin (Figure 6A), C-myc (Figure 6B), KRT6 (Figure 6C) transcript expressions. Likewise, the inhibition of miR-133a-3p and miR-145-5p also evidently diminished β-catenin (Figure 6D, 6E), C-myc (Figure 6D, 6F), KRT6 (Figure 6D, 6G) protein expressions. Altogether, this evidence revealed that miR-133a-3p and miR-145-5p knockdown could repress goat HFSCs differentiation.

### chi-miR-133a-3p and chi-miR-145-5p enhance immunofluorescence intensity of marker-genes of goat HFSCs differentiation

**Figure 7:**
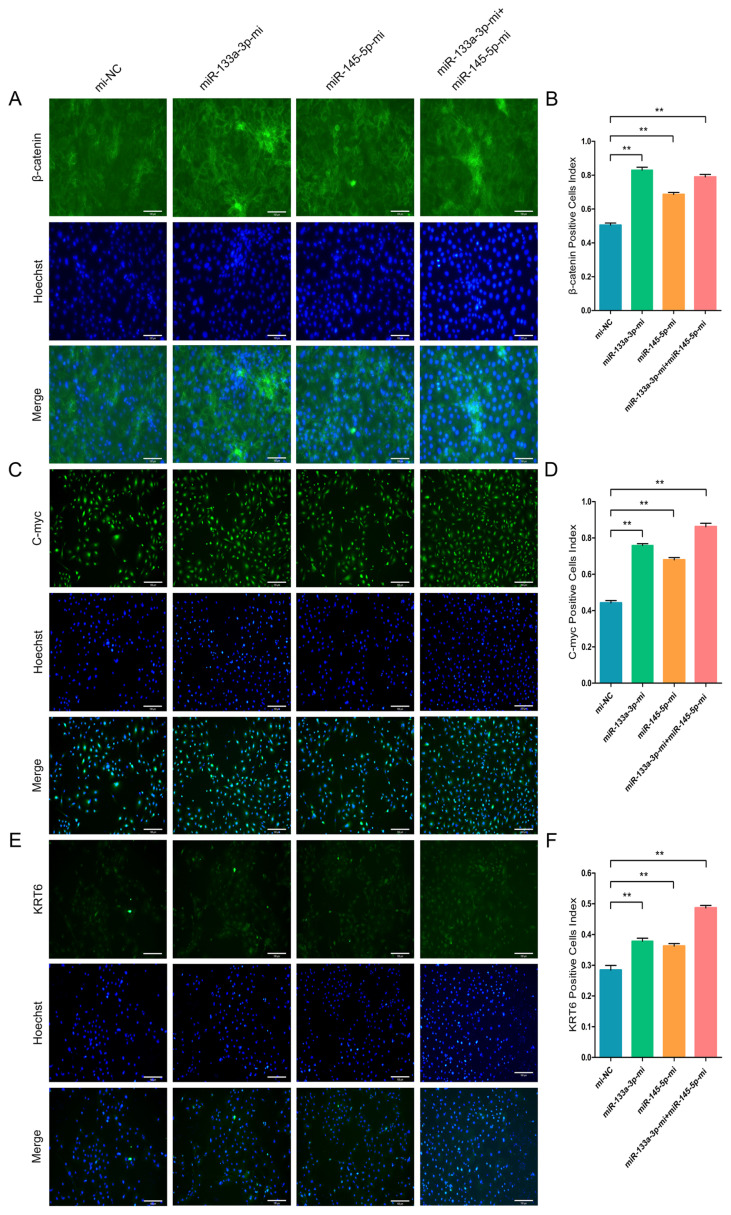
miR-133a-3p and miR-145-5p enhance the immunofluorescence (IF) intensity of differentiation bio-markers in hair follicle stem cells (HFSCs). (A, B) β-catenin protein abundance during HFSCs differentiation, as evidenced via IF assays, 5 days post miR-133a-3p and miR-145-5p mimics (Mc) incorporation. (C, D) C-myc protein abundance during HFSCs differentiation, as evidenced via IF assays, 5 days post miR-133a-3p and miR-145-5p mimics (Mc) incorporation. (E, F) KRT6 protein abundance during HFSCs differentiation, as evidenced via IF assays, 5 days post miR-133a-3p and miR-145-5p mimics (Mc) incorporation. ** p<0.01.

**Figure 8:**
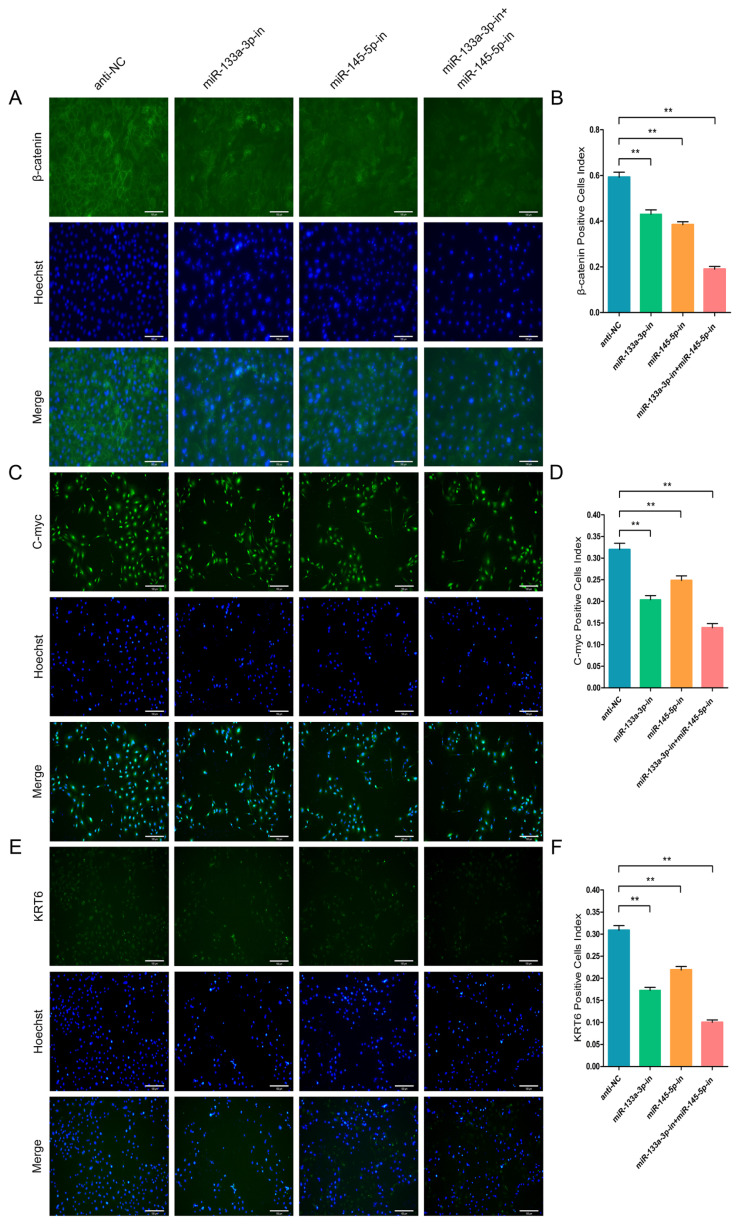
miR-133a-3p and miR-145-5p inhibitors reduce the immunofluorescence (IF) intensity of differentiation bio-markers in hair follicle stem cells (HFSCs). (A, B) β-catenin protein abundance during HFSCs differentiation, as evidenced via IF assays, 5 days following miR-133a-3p and miR-145-5p inhibitors incorporation. (C, D) C-myc protein abundance during HFSCs differentiation, as evidenced via IF assays, 5 days following miR-133a-3p and miR-145-5p inhibitors incorporation. (E, F) KRT6 protein abundance during HFSCs differentiation, as evidenced via IF assays, 5 days following miR-133a-3p and miR-145-5p inhibitors incorporation. ** p<0.01.

To further validate the β-catenin, C-myc, and KRT6 contents in differentiated stem cells, we conducted IF assay. The goat HFSCs IF assay data revealed that miR-133a-3p, miR-145-5p and miR-133a-3p + miR-145-5p separately and obviously enhanced β-catenin (Figure 7A, 7B), C-myc (Figure 7C, 7D), and KRT6 (Figure 7E, 7F) expression. Conversely, miR-133a-3p and miR-145-5p inhibition revealed the aforementioned effects, and reduced β-catenin (Figure 8A, 8B), C-myc (Figure 8C, 8D), and KRT6 (Figure 8E, 8F) expression. Together, our analyses revealed that miR-133a-3p and miR-145-5p could facilitate goat HFSCs differentiation.

## DISCUSSION

HFSCs possess more and more potential characteristics in the progression of multilineage differentiation, such as induced to differentiate into smooth muscle cells, keratinocytes, neurons cells and glial cells [29,30]. The YRDWG HFSCs, which present important roles in SQBH traits synthesis by interacting with its dermal papilla cells. And meanwhile, goat HF development is determined by the stem cell proliferation and differentiation process. Herein, we demonstrated that i) goat HF development and HFSCs growth are regulated by some vital proteins and pathway, namely, β-fibrinogen, β-catenin, keratin family protein and Wnt/β-catenin pathway, MAPK pathway [12]; ii) miR-149-5p could target *CMTM3* gene and then upregulated *AR* gene expression, which emerges an essential role during SQBH synthesis by positively facilitating HFSCs proliferation and differentiation [14,23]; iii) Unlike miR-149-5p, circCOL1A1, a circular RNA which sequesters miR-149-5p, plays negative role in HFSCs proliferation and differentiation [24]; *DUSP6* gene, also called *MKP3* gene, plays critical physiological modulatory functions in MAPK pathway; meanwhile, DUSP6 can be both targeted by miR-133a-3p and miR-145-5p, and miR-133a-3p, and miR-145-5p can suppress HFSCs proliferation via regulation of DUSP6 levels [25,26]. Herein, we further investigated the significance of both miRNAs (miR-133a-3p and miR-145-5p) in goat HFSCs differentiation to extensively reveal the modulatory function of miR-133a-3p and miR-145-5p in goat HFSCs growth.

miR-133a-3p and miR-145-5p, belong to miR-133 family and miR-145 family, both differentially regulated in produced common-quality brush and SQBH goats, and they exerted low contents in the skin tissues of produced common-quality brush hair goats, and showed higher expression levels in the skin tissues of SQBH goats (Supplementary Figure S2A and 2B). This result suggests that high expression levels of miR-133a-3p and miR-145-5p in skin tissue are beneficial to produce SQBH, which also means miR-133a-3p and miR-145-5p may promote goat skin tissue HFSCs differentiation. Therefore, we speculated that the roles of the two miRNAs roles may be comparable to the miR-149-5p role in goat HFSCs differentiation but play converse roles in regulating the stem cell proliferation. In the other types of cells, miR-133a-3p and miR-145-5p also play different roles. Such as, in myogenic cells, miR-133a could promote myogenic cell proliferation and skeletal muscle development by controlling serum response factor (SRF) and transforming growth factor beta receptor 1 (TGFβR1) expression [31]; miR-145-5p could target SRY-box transcription factor 11 (*SOX11*) gene and function in tumor-suppressive roles, then to further mediate MYCN proto-oncogene, bHLH transcription factor (*MYCN*) gene during neuroendocrine differentiation of prostate cancer cells [32]. In mouse, miR-24 could limit and inhibit the intrinsic growth ability of HF progenitor by directly targeting phosphoinositide3-kinase (*PIK3*) gene and reducing the key cycling protein for cell-cycle entry cyclin E1 (CCNE1) expression [33]. During HF development, miR-205 is highly enriched in epithelial progenitors and HFSCs, and functions as a HFSCs activator with a vital role in regulating PI3K pathway and HF morphogenesis [34]. These known research and facts suggested that same or distinct miRNAs serve varied modulatory function in HF development or the other physiological progresses.

NANOG is a recognized gene in keeping and maintaining the totipotency of ESCs. The major role of NANOG is to prevent the ESC differentiation and keep the cells’ totipotency or pluripotency, while the expression level of NANOG need to be downregulated to promote cell differentiation during embryo development [35,36]. SOX9 is an important transcriptional and regulatory factor with an extremely conservative HMG domain structure. *SOX9* gene determines the generation of HFSCs during the early stage of mouse embryo development. Simultaneously, SOX9 also plays an important role in stem cell differentiation [29,37]. Above-mentioned research suggested that *NONOG* and *SOX9* genes are important and play different roles in regulating embryo and HF development. In this research, we demonstrated that miR-133a-3p and miR-145-5p individually or together overexpression in differentiated goat HFSCs decreases NANOG content and increases the expression of SOX9, while individual or together inhibition of miR-133a-3p and miR-145-5p in the differentiated stem cells increases NANOG content, decreases the expression of SOX9. These findings indicated that miR-133a-3p and miR-145-5p might promote goat HFSCs differentiation by reducing NANOG levels and increasing SOX9 expression. In addition, our subsequent stem cell differentiation assays further demonstrated that miR-133a-3p and miR-145-5p individually or together overexpression upregulated β-catenin, C-myc, and KRT6 expressions in goat HFSCs differentiation, whereas miR-133a-3p and miR-145-5p inhibition downregulated the expression level of these stem cell differentiation indicators. This resembles the miR-149-5p function during the differentiation of goat HFSCs [14].

In conclusion, based on our obtained results and these existing studies, we demonstrated that miR-133a-3p and miR-145-5p promote goat HFSCs differentiation by downregulating NANOG expression and upregulating SOX9 and goat HFSCs differentiation bio-markers (β-catenin, C-myc, and KRT6) expression. In addition, these findings could more completely explain the modulatory function of miR-133a-3p and miR-145-5p in goat HFSCs growth, which also provide more understandings or viewpoints for further investigating goat hair follicle development.

## Figures and Tables

**Table 1:** Sequence information for miR-133a-3p and miR-145-5p oligos

Name	Sequence name	Sequence information (5′ to 3′)
miR-133a-3p	miR-133a-3p mimics	UUUGGUCCCCUUCAACCAGCUG (sense)
		GCUGGUUGAAGGGGACCAAAUU (antisense)
	miR-133a-3p inhibitors	CAGUACUUUUGUGUAGUACAA
miR-145-5p	miR-145-5p mimics	GUCCAGUUUUCCCAGGAAUCCCU (sense)
		GGAUUCCUGGGAAAACUGGACUU (antisense)
	miR-145-5p inhibitors	AGGGAUUCCUGGGAAAACUGGAC
mi-NC	Mimics NC	UUCUCCGAACGUGUCACGUTT (sense)
		ACGUGACACGUUCGGAGAATT (antisense)
anti-NC	Inhibitors NC	CAGUACUUUUGUGUAGUACAA

**Table 2:** Primer sequence information for RT-PCR and RT-q-PCR

Gene	Sequence name	Sequence information (5′ to 3′)
*miR-133a-3p*	Stem-loop RT-miR-133a-3p^1)^	GTCGTATCCAGTGCAGGGTCCGAGGTATTCG
		CACTGGATACGACCAGCTGGT
	Stem-loop miR-133a-3p-F	TTTGGTCCCCTTCAACC
	Stem-loop miR-133a-3p-R	GTGCAGGGTCCGAGGT
*miR-145-5p*	Stem-loop RT-miR-145-5p^2)^	GTCGTATCCAGTGCAGGGTCCGAGGTATTCG
		CACTGGATACGACAGGGATTC
	Stem-loop miR-145-5p-F	GTCCAGTTTTCCCAGGA
	Stem-loop miR-145-5p-R	GTGCAGGGTCCGAGGT
*18S-rRNA*	18S-rRNA-F	GTGGTGTTGAGGAAAGCAGACA
*ID:493779*	18S-rRNA-R	TGATCACACGTTCCACCTCATC
*NANOG*	NANOG-F	ACCATCAGGGGTGTTTGGTG
*ID:102178711*	NANOG-R	AGATCCCATACAATAGCCGTCAC
*SOX9*	SOX9-F	GGTGCTCAAGGGCTACGACT
*ID:106503295*	SOX9-R	CCGTTCTTCACCGACTTCCTCC
*β-catenin*	β-catenin-F	TGTTCGCCTTCACTACGGAC
*ID:102191742*	β-catenin-R	TTGCTGGACAAAGGGCAAGA
*C-myc*	C-myc-F	ACGGAACTCTTGCGCCTAAA
*ID:102171262*	C-myc-R	GCCAAGGTTGTGAGGTTGTTC
*KRT6*	KRT6-F	CAGTCCACTGTCTCTGGTGG
*ID:100860930*	KRT6-R	CTGAAGCCACCTCCAATGCT
*GAPDH*	GAPDH-F	AGGTCGGAGTGAACGGATTC
*ID:100860872*	GAPDH-R	CCAGCATCACCCCACTTGAT

RT-PCR, reverse transcription polymerase chain reaction; RT-q-PCR, real-time quantitative polymerase chain reaction; KRT6, Keratin 6; GAPDH, glyceraldehyde-3-phosphate dehydrogenase.

1) Stem-loop RT-miR-133a-3p was applied for reverse transcription (RT) of miR-133a-3p.

2) Stem-loop RT-miR-145-5p was applied for RT of miR-145-5p.

## Data Availability

All data obtained or analyzed in this study are available from the corresponding author upon request.

## SUPPLEMENTARY MATERIAL

**Supplementary Figure S1**. Efficiency of chi-miR-133a-3p and chi-miR-145-5p oligos in differentiated hair follicle stem cells (HFSCs).

**Supplementary Figure S2**. chi-miR-133a-3p and chi-miR-145-5p expression in skin tissue from brush hair goats.
