# Supplementary material for: miR-133a-3p and miR-145-5p co-promote goat hair follicle stem cell differentiation by regulating NANOG and SOX9 expression
Source: Anim Biosci. 2023 Nov 2;37(4):609–21. doi: 10.5713/ab.23.0348 (PMC10915213; doi:10.5713/ab.23.0348)
Supplement: Supplementary file 1 [file ab-23-0348-Supplementary-Fig-S1.pdf]

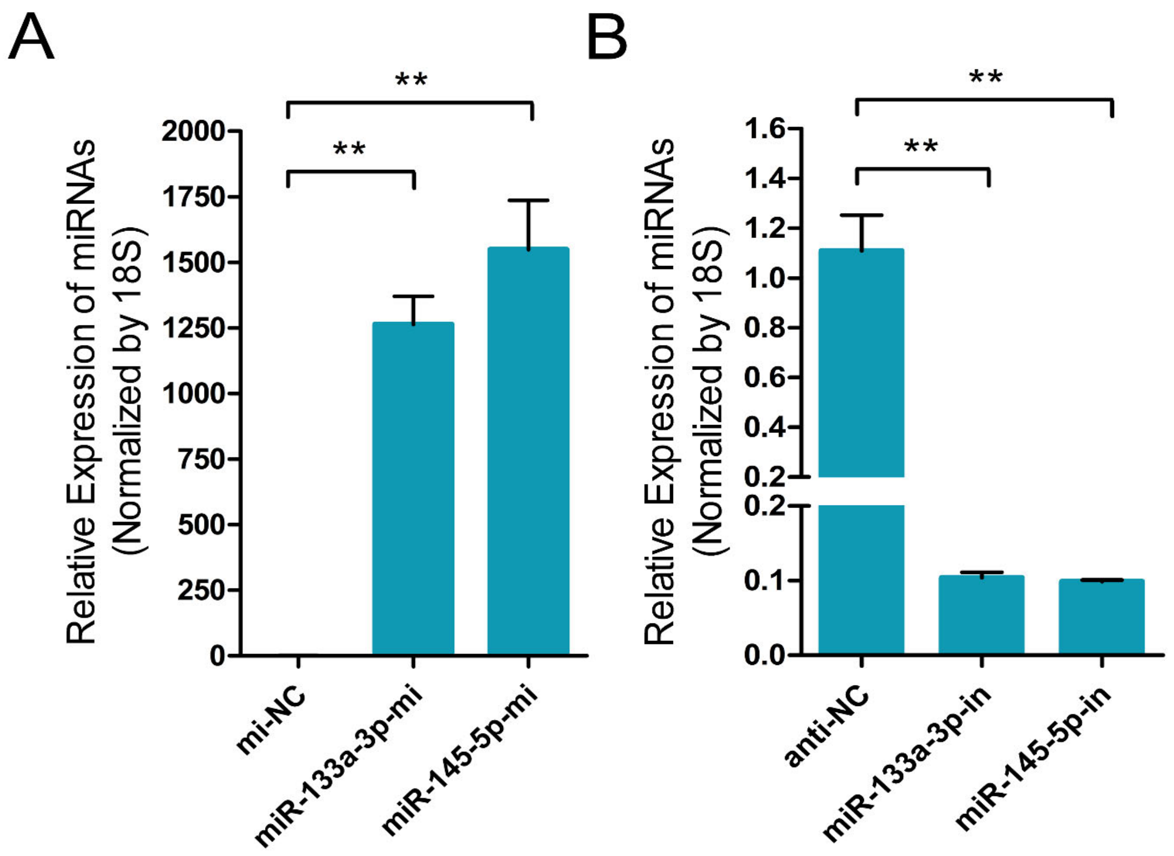

Supplementary Figure S1. Efficiency of chi-miR-133a-3p and chi-miR-145-5p oligos in differentiated hair follicle stem cells (HFSCs). (A) Efficiency of miR-133a-3p and miR-145-5p mimics (Mc). (B) Efficiency of miR-133a-3p and miR-145-5p inhibitors.
