# Supplementary material for: miR-133a-3p and miR-145-5p co-promote goat hair follicle stem cell differentiation by regulating NANOG and SOX9 expression
Source: Anim Biosci. 2023 Nov 2;37(4):609–21. doi: 10.5713/ab.23.0348 (PMC10915213; doi:10.5713/ab.23.0348)
Supplement: Supplementary file 2 [file ab-23-0348-Supplementary-Fig-S2.pdf]

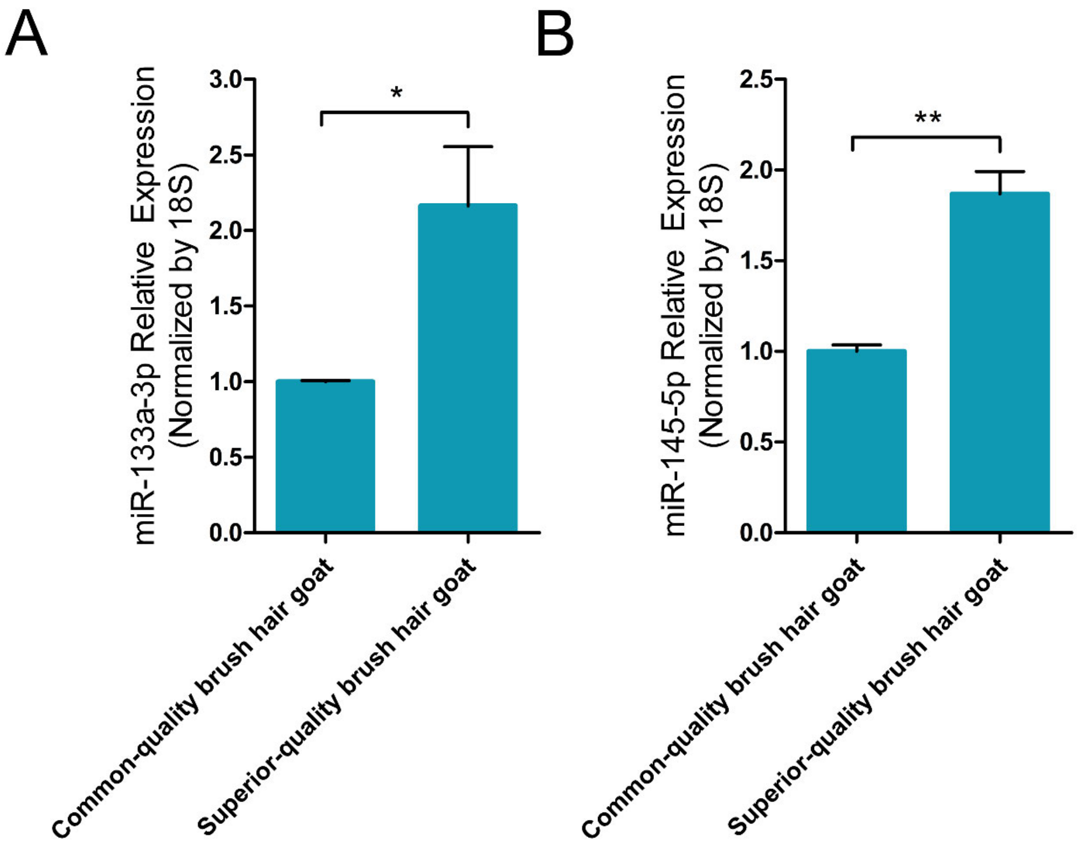

Supplementary Figure S2. chi-miR-133a-3p and chi-miR-145-5p expression in skin tissue from brush hair goats. (A) The difference in miR-133a-3p expression in skin tissue between common-quality brush hair goat and superior-quality brush hair goat. (B) The difference in miR-145-5p expression in skin tissue between common-quality brush hair goat and superior-quality brush hair goat.
